# HBcAb seropositivity is correlated with poor HIV viremia control in an Italian cohort of HIV/HBV-coinfected patients on first-line therapy

**DOI:** 10.1038/s41598-019-46976-1

**Published:** 2019-08-16

**Authors:** Vincenzo Malagnino, Carlotta Cerva, Gaetano Maffongelli, Elisabetta Teti, Luca Foroghi Biland, Novella Cesta, Margherita De Masi, Christof Stingone, Daniele Armenia, Valentina Svicher, Romina Salpini, Massimo Andreoni, Loredana Sarmati

**Affiliations:** 1 Clinic of Infectious Diseases, Policlinico Tor Vergata, Rome, Italy; 2 Department of Systems Medicine, Tor Vergata University of Rome, Rome, Italy; 3 Department of Experimental Medicine and Surgery, Tor Vergata University of Rome, Rome, Italy; 4 UniCamillus, Saint Camillus International University of Health Sciences, Rome, Italy

**Keywords:** Outcomes research, Infectious diseases

## Abstract

The morbidity and mortality rates of human immunodeficiency virus (HIV)-hepatitis B virus (HBV) coinfection are higher than that of either infection alone. Outcomes and the virological response to antiretrovirals (combination antiretroviral therapy, cART) were explored in HIV/HBV subjects in a cohort of Italian patients treated with cART. A single-center retrospective analysis of patients enrolled from January 2007 to June 2018 was conducted by grouping patients by HBV status and recording baseline viro-immunological features, the history of virological failure, the efficacy of cART in achieving HIV viral undetectability, viral blip detection and viral rebound on follow up. Among 231 enrolled patients, 10 (4.3%) were HBV surface (s) antigen (HBsAg)-positive, 85 (36.8%) were positive for antibodies to HBV c antigen (HBcAb) and with or without antibodies to HBV s antigen (HBsAb), and 136 were (58.9%) HBV-negative. At baseline, HBcAb/HBsAb^+/−^-positive patients had lower CD4+ cell counts and CD4+ nadirs (188 cell/mmc, IQR 78–334, p = 0.02 and 176 cell/mmc, IQR 52–284, p = 0,001, respectively). There were significantly higher numbers of AIDS and non-AIDS events in the HBcAb^+^/HBsAb^+/−^-positive subjects than in the HBV-negative patients (41.1% vs 19.1%, p = 0.002 and 56.5% vs 28.7%, respectively, p ≤ 0.0001); additionally, HIV viremia undetectability was achieved a significantly longer time after cART was begun in the former than in the latter population (6 vs 4 months, p = 0.0001). Cox multivariable analysis confirmed that after starting cART, an HBcAb^+^/HBsAb^+/−^-positive status is a risk factor for a lower odds of achieving virological success and a higher risk of experiencing virological rebound (AHR 0.63, CI 95% 0.46–0.87, p = 0.004 and AHR 2.52, CI 95% 1.09–5.80, p = 0.030). HBcAb-positive status resulted in a delay in achieving HIV < 50 copies/mL and the appearance of viral rebound in course of cART, hence it is related to a poor control of HIV infection in a population of coinfected patients.

## Introduction

Due to the introduction of combined antiretroviral therapy (cART), and in particular the introduction of drugs with activity against hepatitis B virus (HBV), such as Lamivudine (LAM), Tenofovir (TDF) and other nucleoside/nucleotide analogues (NA), the ability to control HBV infection has strongly increased in HIV-coinfected patients. However, compared to HIV mono-infected patients, HIV/HBV coinfected patients reach immune-virologic control with more difficulty and have a higher rate of acquired immunodeficiency syndrome (AIDS) events and end-stage liver disease (ESLD) despite the introduction of cART^1,2^. HBsAg-positive cART-treated patients demonstrate impaired CD4 recovery and accelerated evolution towards AIDS^3^ in addition to increased HBV replication, liver disease progression, hepatocellular carcinoma (HCC) prevalence and liver-related mortality^4,5^.

Based on these data, the European Clinical Practice Guidelines for the management of HBV infection^6^ recommended the use of anti-HBV active drugs (NAs) containing cART in HIV-positive patients and strongly suggested avoiding the interruption of this type of treatment. Conversely, no suggestions were provided regarding the management of HIV-positive patients with HBV-resolved infections (i.e., the presence of anti-HBc and/or anti-HBs in the absence of HBsAg). Resolved HBV infection is known to be associated with HBV reactivation in immunocompromised patients subsequent to transplantation or chemotherapy for solid or hematological neoplasia^7^. As a component of resolved infection, occult hepatitis B Infection (OBI), which is defined as the absence of the HBs antigen (HBsAg) in the presence of intrahepatic or plasma HBV replication, is also known to be a risk factor for the evolution of HBV infection in cirrhosis, ESLD and HCC in immunocompromised patients^8^. Very few data are available regarding the influence of resolved HBV infection and OBI on HIV infection control^9,10^, especially in people with low-prevalence HBV infection.

The goal of this study was to explore whether having a resolved HBV infection affects overall health outcomes by comparing a group of cART-treated, HBV/HIV-coinfected Italian subjects to a group of HIV mono-infected patients. Clinical, virological and immunological data of patients seen from January 2007 until July 2018 at the Infectious Diseases Unit of the Policlinico Tor Vergata were retrospectively evaluated.

## Materials and Methods

### Study design

An observational retrospective study of 671 HIV-positive patients collected from January 2007 to July 2018 at the Infectious Diseases Unit of the Policlinico Tor Vergata in Rome, Italy was conducted. Specifically, for this study, a database was built that included all patients’ data at baseline, at the start of cART treatment, and at follow-up visits. In particular, the following data were collected for all of the patients: hematochemical data and HBV and HCV serology at baseline, HIV-RNA viral load, CD4+ cell count at baseline and pre-cART, time since the HIV infection diagnosis and the start time of cART treatment. Data on flares in transaminases (defined as an increase in aspartate aminotransferase (AST) or alanine aminotransferase (ALT) > 50 IU/L), HIV viral blips (VB, defined as a single detection of HIV-RNA > 50 cp/mL), virological rebound (VR, defined as two consecutive HIV-RNA values ≥ 50 cp/ml or one > 1000 cp/mL with consequent changes in cART) and time required to achieve viral undetectability (virological success [VS], defined as two consecutive HIV-RNA < 50 cp/mL) were collected during follow-up examinations. Moreover, data regarding the antiviral treatment for chronic hepatitis B (CHB) (LAM, TDF, Tenofovir Alafenamide [TAF] or Entecavir [ETV]) were also gathered. When available, HBV-DNA viral load data were collected.

No specific ethics committee’s consent was required due to the retrospective nature of the study, which is based on information available from existing clinical documentation [Determination of the Italian Drug Agency (AIFA) of 20 March 2008]. With regard for privacy, all personal information was treated in a confidential manner, and all clinical data were anonymously analyzed.

### Inclusion and exclusion criteria

**Figure 1:**
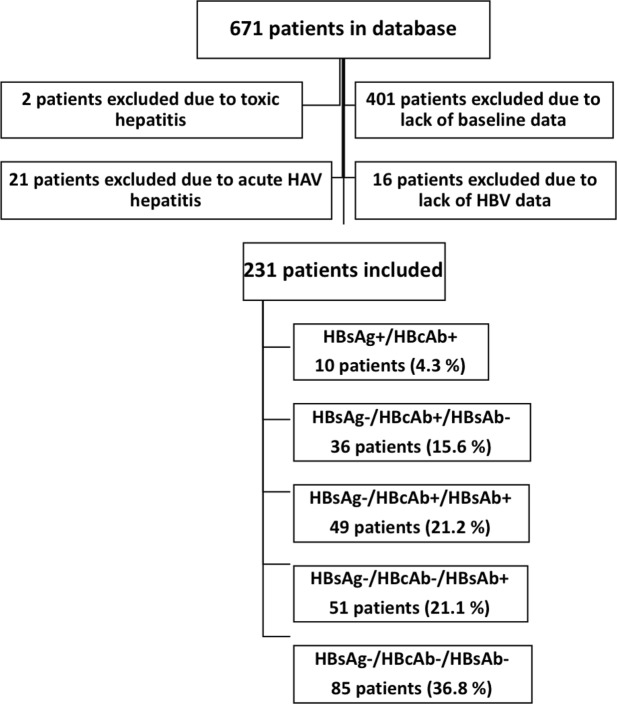
Algorithm of the study population inclusion and exclusion criteria.

As shown in Fig. 1, of the 671 patients followed in our center, 401 were excluded due to a lack of virological or immunological data at the time of diagnosis (including patients who were non-adherent to follow-up visits, who transferred from other clinical centers and who voluntarily stopped first-line cART treatment), and 16 patients were excluded because of the loss of HBV serology data before the start of ART. Twenty-three patients were excluded due to the presence of transaminase flares dependent on hepatic drug toxicity (2 patients) or acute hepatitis A virus (HAV) infection (21 patients), which would invalidate the evaluation of transaminase flares. Two hundred thirty-one patients were ultimately studied.

### Endpoints

The primary endpoint of this study was to investigate the influence of HBcAb^+^/HBsAb^+/−^ status on time required to achieve an HIV viral load < 50 copies/mL after the initiation of cART and the onset of VB or VR after HIV VS. Subsequently, the effects of HBV antigen/antibody profiles (i.e., HBsAg, HBcAb and/or HBcAb/HBsAb positivity) on overall mortality and the appearance of AIDS-related events were also evaluated.

Furthermore, non-AIDS events, including hypertension, diabetes and lipidic disorders, bone disorders (osteopenia, osteoporosis), renal impairment and non-AIDS-related neoplastic pathologies, were investigated.

### Laboratory testing for the diagnosis of HBV and HIV infection

HBV serological markers were measured using immune-enzymatic assays (Roche/Cobas Diagnostics, Rotkreuz, Switzerland). Plasma HBV-DNA was identified using real-time polymerase chain reaction (lower limit of quantification: 20 IU/ml) (Roche/Cobas Ampliprep/Cobas Taqman, Rotkreuz, Switzerland). Plasma HIV-RNA levels were measured using a commercial test, with 20 copies/ml of HIV-RNA defined as the lower limit of quantification (COBAS AmpliPrep/COBAS TaqMan HIV-1 Test, v2.0).

### Statistical methods

All statistical analyzes were conducted using STATA 14.2 (College Station, TX).

The study population is described using proportions and percentages for categorical values and median measurements and interquartile ranges (IQRs) for continuous values. The comparison between HBcAb^+^/HBsAb^+/−^-positive and HBV-negative patients was performed with the Kruskal-Wallis test for continuous variables and with the Chi-squared test or Fisher’s exact test, when appropriate, for categorical variables.

Kaplan-Meier curves were used to estimate the time required to achieve and the probability of achieving VS after the start of cART and the probabilities of experiencing VB and VR in the HBcAb^+^/HBsAb^+/−^-positive and HBV-negative groups of patients.

Cox regression analysis was performed to evaluate the association between HBcAb and the risk of achieving VS or experiencing VR after controlling for other potential confounding factors under the assumption of proportionality of the hazards. The following variables were considered potential confounders: age, AST levels, hepatitis C virus (HCV) coinfection, pre-cART plasma HIV-RNA, pre-cART CD4 count, anti-HBV treatment, risk factor for HIV acquisition, calendar year, and type of cART.

## Results

### Description of the study population

In Table 1, the characteristics of the study population are reported: 231 patients were included in the study, the median age was 42 years old (IQR 32–52), 75% were males, and 200 patients (86.6%) had acquired HIV through a sexual relationship. The median baseline HIV viral load was 85.605 cp/mL (IQR 31.199–285.442), and the median baseline CD4+ cell count was 236.5 (IQR 100–410). The patients were followed for a median duration of 59.4 months (4.9 years) (IQR 35.5–88.4). Regarding the serological status of HBV infection, ten (4.3%) of patients were HBsAg-positive, and 85 (36.8%) were HBcAb-positive/HBsAg-negative; among these, 36 (15.6%) were HBsAb-negative, and 49 (21.2%) were HBsAb-positive. One hundred thirty-six patients (58.9%) were HBV-negative, and 51 (22.1%) of these patients had received prior vaccinations.

**Table 1:** Study population characteristics.

Total study population, patients, n.	231
Age, years, median (IQR)	42 (32–52)
Sex ratio, M/F (M%)	175 (75.5%)
**Origin**, **n (%)**	
-Italian	183 (79.2%)
-European	13 (56%)
-African	31 (13.4%)
-Other	4 (1.7%)
**Risk factors**	
-Sexual, n (%)	200 (86.6%)
-IDUs, n. (%)	31 (13.4%)
Months from cART initiation, median (IQR)	1,5 (0.4–4)
Duration of cART, months, median (IQR)	50,2 (32.7–82.8)
Follow-up, months, median (IQR)	59,4 (35.5–88.4)
AST, UI/L, median (IQR)	22 (1.34)
ALT, UI/L, median (IQR)	29 (23–43)
Platelet count, 10^6 cells/µL, median (IQR)	196 (137–248)
Transaminases flare, median (IQR)	115 (49.8%)
FIB-4 at baseline, median (IQR)	0,87 (0.57–1.50)
CD4+ at baseline, cells/mmc, median (IQR)	236,5 (100–410)
HIV-RNA VL at baseline, cp/mL median (IQR)	85.605 (31.199–285.442)
Undetectability after 6 months of cART, Yes/No, n. (%)	171 (74.3%)
Undetectability, months, median (IQR)	5 (3-.,5)
AIDS-related event, Yes/No, n. (%)	64 (27.7%)
Non-AIDS related event, Yes/No, n. (%)	90 (38.9%)
HIV mono-infected people, patients, n.	136
Vaccinated for HBV (antiHBc-negative/anti-HBs-positive), n.	51
Not vaccinated (antiHBc-negative/anti-HBs-negative), n.	85
Coinfected HIV/HBV, patients, n.	95
-HBsAg+/AntiHBc+, n. (%)	10 (4.3%)
-AntiHBc+/AntiHBs−, n. (%)	36 (15.6%)
-AntiHBc+/AntiHBs+, n. (%)	49 (21.2%)
Anti-HCV+, n. (%)	13 (5.6%)
-HCV-RNA positive, n. (%)	3 (1.2%)
**cART strategy**, **n**. **(%):**	
-Lamivudine	38 (16.4%)
-Tenofovir/TAF	176 (76.2%)
-No anti-HBV therapy	17 (7.4%)
>1 year without anti-HBV agent, Yes/No	23 (10%)
HIV VR, n. (%)	34 (14.8%)
HIV VB, n. (%)	101 (44.1%)
Deaths, n. (%)	10 (4.3%)

cART: combined antiretroviral therapy; AST: aspartate aminotransferase; ALT: alanine aminotransferase; Undetectability: HIV-RNA < 50 copies/mL VR: virological rebound; VB: HIV viral blips.

One hundred seventy-one patients (74.3%) achieved HIV viremia < 50 copies/mL within a median of 5 months (IQR 3–6.5). The large majority (92.6%) of patients were treated with cART-containing anti-HBV agents: 38 (16.4%) with LAM and 176 (76.2%) with TDF or TAF. Overall, during the observation period, AIDS-related events were reported in 64 (27.7%) patients, and 90 (38.9%) developed non-AIDS-related events. HIV VR occurred in 34 (14.8%) subjects, and 101 (44.1%) showed occasional HIV VB during the follow-up period.

#### HBsAg-positive patients

Ten (4.3%) out of 231 patients were HBsAg-positive; these patients had a median pre-cART CD4 cell count of 193 cell/mmc (IQR 98-373) and an HIV viral load of 87.100 copies/mL (IQR 31.100–143.000). All had detectable plasma HBV-DNA (median 9.260 UI/L, IQR 2.651–69.300 UI/L) and were taking cART: 2 (20%) as a regimen containing LAM and 8 (80%) as a regimen containing TDF or TAF. Of these 10 patients, 2 (20%) died because of HCC, 1 (10%) had HCC treated with chemoembolization, 2 (20%) developed an AIDS-related event (2 cases of *Pneumocystis jirovecii* pneumonia [PJP]), and another 3 patients (30%) developed a non-AIDS-related event (2 cases of cardiovascular disease and 1 of diabetes complications) (data not shown).

#### Comparison between HBcAb^+^/HBsAb^+/−^-positive and HBV-negative patients

A comparison between HBV-negative and HBcAb^+^/HBsAb^+/−^ HIV-positive patients is reported in Table 2. Compared to HBV-negative subjects, HBcAb^+^/HBsAb^+/−^-positive patients were older (median, 48 years old [39–55] vs 39 years old [29–48], p 0.0001) and more likely to have a history of intravenous drug use (17 [54.8%] vs 11 [35.5%], p 0.01). Compared to HBV-negative patients, HBcAb^+^/HBsAb^+/−^-positive patients had more frequent episodes of transaminase flares (56 [65.9%] vs 49 [36%], p < 0.0001), significantly lower CD4+ cell counts at baseline (188 [IQR 78–334] vs 293 [IQR 127–443], p = 0.02), and lower pre-cART CD4+ cell counts (176 [IQR 52–284] vs 239 [IQR 127–443], p = 0.02).

**Table 2:** Comparison between HBcAb/HBsAb^+/−^-positive vs HBV-negative patient characteristics.

Study population	HBcAb/HBsAb^+/−^-positive (n = 85)	HBV-negative (n = 136)	P-value
Age, years, median (IQR)	48 (39–55)	39 (29–48)	**0.0001**
**Risk factors**, **n**. **(%)**			
Sexual	68 (34%)	125 (62.5%)	**0.01**
IDUs	17 (54.8%)	11 (35.5%)	**0.01**
Months from cART initiation, median (IQR)	1.3 (0.6–4.9)	1.4 (0.85–3.6)	0.25
Duration of cART, months, median (IQR)	49.8 (29.3–84.7)	49.4 (33.9–79)	0.89
Calendar years since cART start, median (IQR)	2013 (2011–2015)	2013 (2011–2015)	0.58
Follow-up, months, median (IQR)	61.9 (34.8–92.3)	56.3 (36.2–84.3)	0.56
AST, UI/L, median, (IQR)	23 (18–34)	21 (16–31.5)	0.40
ALT, UI/L, median, (IQR)	32 (24–43)	28 (21.5–40)	**0.02**
Platelet count, 10^6^/µL median (IQR)	191 (126–227)	200 (164–256)	**0.02**
Flair of transaminases, median (IQR)	56 (65,9%)	49 (36%)	**<0.0001**
FIB-4 at baseline, median (IQR)	1.12 (0.68–1.81)	0.76 (0.48–1.2)	**0.001**
Anti-HCV+, n. (%)	7 (8.2%)	5 (3.6%)	0.23
HCV-RNA-positive, n. (%)	1 (1.1%)	1 (0.7%)	nc
CD4+ at baseline, cell/mmc, median (IQR)	188 (78–334)	293 (127–443)	**0.02**
Pre-cART CD4+ cell/mmc, median (IQR)	176 (52–284)	239 (97–390)	**0.01**
Pre-cART HIV viremia, cp/mL median (IQR)	109.8 (45.6–330.7)	63 (22.4–241.1)	0.14
Undetectability at 6^th^ month, median (IQR)	50 (59.5%)	115 (84.6%)	**<0.0001**
Undetectability, months, median (IQR)	6 (4–8)	4 (3–6)	**0.0001**
AIDS-related events, Yes/No, n. (%)	35 (41,1%)	26 (19,1%)	**0.002**
Non-AIDS related events, Yes/No, n. (%)	48 (56.5%)	39 (28.7%)	**<0.0001**
**cART anti-HBV active drugs**, **n**. **(%):**			
-Lamivudine	14 (16.4%)	22 (16.1%)	0.95
-Tenofovir/TAF	62 (72.9%)	106 (77.9%)	0.66
-No anti-HBV agent	9 (10.6%)	8 (5.9%)	0.28
**Type of cART**			
*PI* + *2 NRTIs*	54 (39.7)	42 (49.4)	0.157
*NNRTI* + *2 NRTI*	46 (33.8)	25 (29.4)	0.494
*INI* + *2 NRTI*	31 (22.8)	12 (14.1)	0.113
*Other*	5 (3.7)	6 (7.1)	0.342
>1 year without anti-HBV agent, n. (%)	12 (14.3%)	11 (8.1%)	0.18
HIV VR, median (IQR)	18 (21.7%)	14 (10.3%)	0.06
HIV VB, median (IQR)	62 (74.7%)	30 (22.1%)	**<0.0001**
Deaths, n.(%)	6 (7.1%)	2 (1.5%)	**0.006**

IDU: injecting drug users; cART: combined antiretroviral therapy; AST: aspartate aminotransferase; ALT: alanine aminotransferase; Undetectability: HIV-RNA < 50 copies/mL; TAF: Tenofovir alafenamide; VR: virological rebound; VB: HIV viral blips.

Once cART was initiated (89% of HBcAb^+^/HBsAb^+/−^-positive patients had an NA in their cART composition), HBV-negative patients achieved HIV-RNA undetectability in a significantly shorter time than was found in HBcAb^+^/HBsAb^+/−^-positive subjects (median time, 4 [IQR 3–6] vs 6 months [IQR 4–8], p < 0.0001). A significantly higher number of AIDS-related and non-AIDS-related events were detected in HBcAb^+^/HBsAb^+/−^-positive subjects than in HBV-negative patients (35 [41.5%] vs 26 [19.1%] and 48 [56.5%] vs 39 [28.7%], p 0.002 and p < 0.0001, respectively). Among HBcAb^+^/HBsAb^+/−^ patients, the following AIDS- and non-AIDS-related events were documented: PJP (16 cases), Kaposi Sarcoma (KS) (7 cases), esophageal candidiasis (4 cases), wasting syndrome (4 cases), PJP plus esophageal candidiasis (1 case), non-AIDS tumors (12 cases), lipidic disorders (19 cases), osteoporosis (6 cases), diabetes (5 cases) and more than one non-AIDS disease (7 cases) (data not shown). HBV-negative patients had the following AIDS and non-AIDS pathologies: PJP (14 cases), KS (2 cases), cerebral lymphoma (1 case), esophageal candidiasis (7 cases), more than one of the above-listed AIDS events (2 cases), non-AIDS tumors (7 cases), lipid disorders (13 cases), osteoporosis (2 cases), hypertension (9 cases) or more than one of the previously mentioned non-AIDS comorbidities (8 cases) (data not shown).

At the end of the follow-up period (the duration of which did not differ between HBcAb^+^/HBsAb^+/−^-positive and HBV-negative patients [p = 0.56]), 62 (74.7%) of the HBcAb^+^/HBsAb^+/−^-positive and 30 (22.1%) of the HBV-negative subjects developed HIV VB (p < 0.0001). Moreover, HBcAb^+^/HBsAb^+/−^ patients were more prone to VR onset (18 [21.7%] vs 30 [22.1%], p < 0.0001). Virological control of HIV during follow-up was poorer in the HBcAb^+^/HBsAb^+/−^ group. Overall, at the end of the follow-up period, 8 out of 221 patients had died. Of these, six (7.1%) were HBcAb-positive, and only two (1.5%) were HBV-negative (p 0.006).

#### Comparison between HBcAb-positive patients with and without HBsAb

In Table 3, the differences between the groups of HBcAb-positive patients with and without HBsAb are evaluated. While no other significant differences were found between these two groups of subjects, among HBcAb-positive patients, HIV VR was found in a significantly higher number of patients without HBsAb than in those with HBsAb (12 [35.3%] vs 6 [12.2%], p = 0.01).

**Table 3:** Comparison between HBcAb/HBsAb-positive vs. HBcAb-positive/HBsAb-negative.

Study population	HBcAb^+^/HBsAb+ (n = 49)	HBcAb^+^/HBsAb−. (n = 36)	P-value
Age, years, median (IQR)	48 (39–55)	47 (39–55)	0,71
Duration of cART, months, median (IQR)	70 (34–96,5)	56 (34–88)	0,73
AST, UI/L, median (IQR)	21 (17–28)	25 (20–45)	0,04
ALT, UI/L, median (IQR)	31 (26–42)	33 (22,5–53)	0,93
Platelet count, 10^6/µL, median (IQR)	192 (127–226)	176 (119–238)	0,91
Flair of transaminases, median (IQR)	30 (61%)	26 (72%)	0,35
HCV-RNA-positive, n. (%)	0 (0)	1 (2,7%)	nc
Baseline CD4+, cell/mmc, median (IQR)	193 (83–363)	159 (58–280)	0,25
Pre-cART CD4+ cell/mmc, median (IQR)	180 (68–302)	159 (45–243)	0,31
Pre-cART HIV cp/mL median (IQR)	102765 (41600–198989)	355996 (49091–405087)	0,24
Undetectability at 6^th^ month, median (IQR)	32 (65,3%)	18 (51,4%)	0,26
AIDS-related events, Yes/No, n. (%)	17 (34,7%)	18 (50%)	0,15
Non-AIDS related events, Yes/No, n. (%)	31 (63,2%)	17 (47,2%)	0,14
**Antiretroviral drugs**, **n**. **(%):**			
-Lamivudine	7 (19,4%)	7 (14,3%)	0,36
-Tenofovir/TAF	37 (75,5%)	25 (69,4%)	0,35
-No anti-HBV agent	5 (10,2%)	4 (11,1%)	NC
>1 year without anti-HBV agent, n. (%)	7 (14,3%)	5 (14,3%)	NC
HIV VR, median (IQR)	6 (12,2%)	12 (35,3%)	**0,01**
HIV VB, median (IQR)	34 (69,4%)	28 (82,3%)	0,18

IDU: injecting drug users; cART: combined antiretroviral therapy; AST: aspartate aminotransferase; ALT: alanine aminotransferase; TAF: Tenofovir alafenamide; VR: virological rebound; VB: HIV viral blips.

#### HIV virological response to cART in HBcAb-positive and -negative patients

**Figure 2:**
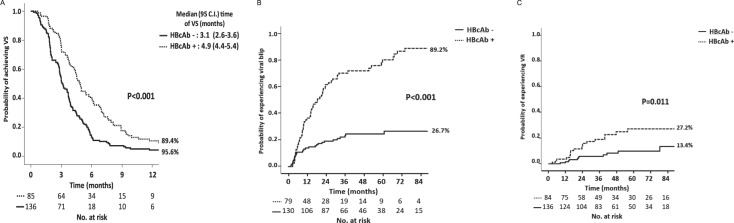
(**A**) KM estimates of the odds of VS (virological success, HIV < 50 copies/mL) according to HBcAb serostatus; (**B**) KM estimates of VB (viral blips) after the achievement of VS according to HBcAb serostatus; (**C**) KM estimates of the odds of experiencing VR (virological rebound) after the achievement of VS according to HBcAb serostatus.

KM estimates were produced to analyze the ability to reach the VS after the start of first-line cART and the risk of incurring VR after virological success in the 2 groups of HBcAb-positive and -negative HIV patients. In Fig. 2, Kaplan-Meier curves show that, by 12 months after cART start, HBcAb-positive patients had a longer median time and a lower probability of achieving VS compared to HBcAb-negative patients (89.4% vs 95.6%, p < 0.001) (Panel A). Concerning the probability of experiencing a VB, Kaplan-Meier estimates showed that by the 84^th^ month after the achievement of VS, HBcAb-positive patients had a higher probability of experiencing a VB compared to HBcAb-negative patients (26.7% vs 89.2%, p < 0.001) (Fig. 2, Panel B).

Finally, concerning VR, in Fig. 2 panel C, it is shown that by the 84^th^ month after the achievement of VS, HBcAb-positive patients had a higher probability of experiencing a VR compared to HBV-negative patients (13.4% vs 27.2%, p = 0.011). Patients who experienced VR had a higher number of blips (median number of VB 2 [IQR 1–3]) compared to those who did not experience VR (median number of VB 0 [IQR 0–1]) (p < 0.001 Mann-Whitney test) (data not shown).

Therefore, Kaplan Meier estimates showed that antiHBc-positive patients exhibited less virological control from the beginning of cART, including a delayed VS and subsequently more frequent occurrence of VB and VR.

Table 4 shows the adjusted hazard ratio (AHR) of achieving VS and experiencing VR after the start of cART. The following potential confounders were considered: age, AST levels, HCV coinfection, pre-cART plasma HIV-RNA, pre-cART CD4 count, anti-HBV treatment, risk factor for HIV acquisition, calendar year, and type of cART. HBcAb positivity, either with or without HBsAb, was associated with a lower AHR of achieving VS (AHR 0.63, CI 95% 0.46–0.87, p = 0.004) and an increased risk of VR after cART beginning (AHR 2.52, CI 95% 1.09–5.80, p = 0.030). After adjustment for potential confounders, HBcAb-positive status was confirmed to be a risk factor for lower HIV virological control after the initiation of first-line cART. Moreover, patients with a high pre-cART HIV viral load had a lower AHR of achieving VS (HIV-RNA > 100.000 and <500.000 cp/mL, AHR 0.68, CI 95% 0.47–0.98, p = 0.038; and HIV-RNA > 500.000 cp/mL AHR 0.40, CI 95% 0.25–0.66, p < 0.001), and patients with a pre-cART HIV-RNA > 500.000 cp/mL had a higher AHR of VR than was found in those with a lower level of viremia (AHR 4.99, CI95% 1.42–17.55, p = 0.012). The presence of integrase inhibitors (INIs) plus 2 nucleos(t)ide reverse transcriptase inhibitors (NRTIs) in the cART regimen was associated with a higher AHR of achieving VS (AHR 1.85, CI 95% 1.20–2.85, p = 0.006) as was cART initiation in a more recent calendar year (AHR 1.11, CI 95% 1.05–1.18, p < 0.001).

**Table 4:** Cox regression models for estimated factors with predictive impact on VS and VR.

	Risk of achieving VS				Risk of experiencing VR			
	AHR^a^	95.0% C.I.		P value	AHR^a^	95.0% C.I.		P value
		Lower	Upper			Lower	Upper	
Age (per 5 years higher)	1.05	0.98	1.12	0.143	0.85	0.71	1.01	0.057
AST elevated x2N	1.05	0.42	2.62	0.914	2.04	0.21	20.12	0.540
HCV coinfection	1.16	0.57	2.36	0.684	2.48	0.40	15.17	0.327
**Plasma HIV-RNA at therapy start (copies/mL)**								
<*100*,*000*^b^	1							
*100*,*000–500*,*000*	0.68	0.47	0.98	**0.038**	2.04	0.74	5.63	0.166
>*500*,*000*	0.40	0.25	0.66	**<0.001**	4.99	1.42	17.55	**0.012**
**CD4 cell count at cART start (cells/mm** ^**3**^ **)**								
<200^b^	1							
*201–350*	1.20	0.83	1.74	0.331	0.24	0.08	0.78	**0.017**
*351–*500	1.07	0.67	1.71	0.767	0.18	0.02	1.47	0.110
>*500*	1.41	0.82	2.45	0.216	0.00	0.00	ND	0.973
Drug abuser as risk factor	0.83	0.51	1.35	0.457	1.02	0.28	3.72	0.980
Anti-HBc^+^	0.63	0.46	0.87	**0.004**	2.52	1.09	5.80	**0.030**
**Anti-HBV drug included in cART**								
*None* ^b^	1							
*TDF*	1.43	0.74	2.75	0.290	0.79	0.16	4.02	0.778
*3TC*	1.83	0.94	3.54	0.074	0.80	0.16	3.99	0.783
Calendar year of cART start	1.11	1.05	1.18	**<0.001**	1.19	0.99	1.43	0.063
**Type of cART**								
*PIb* + *2 NRTIs*^b^	1							
*NNRTI* + *2 NRTI*	1.17	0.84	1.64	0.356	2.74	0.98	7.61	0.054
*INI* + *2 NRTI*	1.85	1.20	2.85	**0.006**	1.38	0.38	5.06	0.627
*Other*	1.25	0.54	2.91	0.606	2.19	0.40	12.00	0.365

^a^Adjusted for age, AST levels, HCV coinfection, pre-ART plasma HIV-RNA, pre-ART CD4 count, anti-HBV treatment, risk factor, calendar year, and type of ART. ^b^Reference (dummy). Bold variables were significantly associated with virological response (p < 0.05). VS: virological success; VR: virological rebound; 3TC: lamivudine; AST: alanine aminotransferase; ART: antiretroviral therapy; INI: integrase inhibitor; NRTI: nucleos(t)ide reverse transcriptase inhibitor; NNRTI: Non-NRTI; PIb: ritonavir/cobicistat boosted protease inhibitor.

## Discussion

In our cohort of HIV-positive patients who started cART, the presence of serological markers of a past HBV infection was significantly correlated with a delay in achieving HIV viremia undetectability and the appearance of HIV VR after the start of cART (AHR 2.52, CI 95% 1.09–5.80, p = 0.030 and AHR 0.63, CI 95% 0.46–0.87, p 0.004, respectively). Moreover, significantly higher numbers of AIDS and non-AIDS-related events were found in the group of HIV/HBcAb-positive patients than in HIV mono-infected individuals.

In our population, 41% of the 231 patients included in this study had an ongoing or resolved HBV infection. Slightly lower rates of HBV-resolved infection or CHB were found in two American cohorts, which reported values of 23% and 35% in HIV seroconverters and HIV patients on cART, respectively^11,12^.

CHB in HIV-positive subjects has been demonstrated to worsen the prognosis of both infections, and a higher risk of mortality was recently observed in patients coinfected with CHB and an HBV DNA viral load > 2000 UI/ml^13^. Very little is known about the influence of resolved HBV infection on the course of HIV infection. From the results of a longitudinal study performed in a large cohort of HIV-negative and -positive patients^7^, the authors concluded that the isolated presence of HBcAb was a sign of an impaired immune response in patients with an HIV infection.

In our study cohort, HBcAb^+^/HBsAb^+/−^-positive subjects presented flares of transaminases more frequently than was found in HBV-negative patients (65.9% vs 36%, p < 0.0001), suggesting the potentially transient reactivation of HBV. Hepatitis flares have been widely documented in HBcAb-positive patients during immunosuppressive therapy^7^, whereas the association of liver diseases with signs of resolved HBV infection in coinfected HIV people is difficult to ascertain as few studies have assessed the association between liver damage and both isolated anti-HBc patterns and occult HBV infections^14^. In the Veterans Aging Cohort Study^15^, the presence of HBcAb was associated with more advanced hepatic fibrosis in HIV-HCV coinfected patients, while Morsica and his colleagues^9^ showed that 21% of HBcAb-positive HIV-coinfected patients in the Italian national cohort (ICONA) presented detectable HBV-DNA associated with an increase in transaminase levels.

An increased number of AIDS and non-AIDS events were documented in the follow-up of our study cohort, which is associated with ongoing or resolved HBV infections. A similar result was reported by Chun HM and his collaborators^12^, who observed that the risk of AIDS and death was high in both CHB and resolved HBV subjects from a cohort of 2536 HIV-positive cART recipients. Similarly, HBV coinfection negatively impacted patient survival in a group of HIV seroconverters, among whom the rates of AIDS and death were significantly higher in CHB patients and increased, although not significantly, in patients with resolved HBV infections^11^.

A series of potential factors have been shown to form the basis of the HBV and HIV interaction and to result in the acceleration of both infection courses^16^. HIV exerts direct activity on hepatocytes and Kupffer cells, thereby contributing to liver inflammation. Moreover, the rate of microbial translocation was higher in HIV/HBV-coinfected individuals than in HBV mono-infected individuals^17^. Microbial translocation has been associated with ongoing immune dysfunction and contributes to the persistence of chronic immune activation and inflammation, which have been associated with the development of comorbidities in HIV-infected persons, even those receiving effective cART^18,19^.

In our study cohort, virological control was worse in HIV/HBcAb/HBsAb^+/−^ patients than in HIV mono-infected subjects in a multivariate analysis, and the status of the resolved HBV coinfection was strongly correlated with HIV VB onset in HIV monoinfected people (89.2% vs 26.7%, p < 0.001).

The appearance of VB is commonly described in cART-treated HIV patients^20^, and some authors have found a correlation between VB and subsequent VR^21–23^. In our study, we found that the median VB was higher in patients with VR than in those without VR. The origin of VB remains uncertain, but an increased HIV replication rate has been correlated with the presence of other infections and with certain vaccinations^24,25^. A number of viral coinfections, when present in HIV-positive subjects, seem to contribute to unfavorable outcomes. Cytomegalovirus coinfection was associated with HIV disease progression^26,27^, and hepatitis C virus is a leading cause of non-AIDS-related mortality among HIV-coinfected patients^28^.

HBV DNA is less rarely detected among patients with HIV and those with isolated anti-HBc (OBI patients), with its rate reported to vary from 0.6% to 89% in several studies^29^, and is related to a poor immune status^8^. Therefore, it is possible to argue that occasional, although limited, HBV replications may also contribute to the appearance of HIV viremia during the course of cART.

Before any conclusions can be drawn, some of the limitations of our study need to be discussed. First, the lack of HBV-DNA data in our population was a major limitation. Currently, no shared indications are used in the monitoring of HBV viremia in coinfected patients with resolved HBV infections, but it may be interesting to ascertain whether poor control of HIV viremia during cART is correlated with the simultaneous presence of HBV-DNA in patients with resolved HBV infections. Second, all of the evaluated patients were included from a single institution, and this might reduce the generalizability of our conclusions. Third, the retrospective nature of this study limits the power of the obtained results.

In conclusion, during the course of cART, an HBcAb-positive status was a risk factor delayed achievement of HIV < 50 copies/mL and the appearance of VR in our cohort of HIV/HBV-coinfected patients. Further studies are needed to assess how HBV-resolved infections exert a harmful effect on HIV and how they might reduce HIV replication control.
